# The importance of accurate muscle modelling for biomechanical analyses: a case study with a lizard skull

**DOI:** 10.1098/rsif.2013.0216

**Published:** 2013-07-06

**Authors:** Flora Gröning, Marc E. H. Jones, Neil Curtis, Anthony Herrel, Paul O'Higgins, Susan E. Evans, Michael J. Fagan

**Affiliations:** 1 Department of Engineering, Medical and Biological Engineering Research Group, University of Hull, Hull HU6 7RX, UK; 2 Research Department of Cell and Developmental Biology, University College London, London WC1E 6BT, UK; 3 Département d'Ecologie et de Gestion de la Biodiversité, Muséum National d'Histoire Naturelle, Case postale 55, Paris Cedex 5 75231, France; 4 Centre for Anatomical and Human Sciences, Hull York Medical School, University of York, York YO10 5DD, UK

**Keywords:** bite force, multi-body dynamics analysis, skull, feeding, validation, *Tupinambis*

## Abstract

Computer-based simulation techniques such as multi-body dynamics analysis are becoming increasingly popular in the field of skull mechanics. Multi-body models can be used for studying the relationships between skull architecture, muscle morphology and feeding performance. However, to be confident in the modelling results, models need to be validated against experimental data, and the effects of uncertainties or inaccuracies in the chosen model attributes need to be assessed with sensitivity analyses. Here, we compare the bite forces predicted by a multi-body model of a lizard (*Tupinambis merianae*) with *in vivo* measurements, using anatomical data collected from the same specimen. This subject-specific model predicts bite forces that are very close to the *in vivo* measurements and also shows a consistent increase in bite force as the bite position is moved posteriorly on the jaw. However, the model is very sensitive to changes in muscle attributes such as fibre length, intrinsic muscle strength and force orientation, with bite force predictions varying considerably when these three variables are altered. We conclude that accurate muscle measurements are crucial to building realistic multi-body models and that subject-specific data should be used whenever possible.

## 1. Introduction

Multi-body dynamics analysis (MDA) is a computer-based simulation method that offers many possibilities for the study of the mechanics of complex, integrated systems such as the vertebrate feeding system. It allows simulation of the forces produced by the masticatory muscles, the resulting bite forces and the reaction forces at the joints, as well as the movement of the jaws [1–3]. To date, MDA has been used in studies of skull mechanics in several species, living and extinct [4–10]. However, to be confident in the results of MDA, models need to be validated against *in vivo* data such as measurements of bite force, jaw motion and muscle activity. Although MDA models are becoming increasingly common in the field of skull mechanics, relatively few studies have directly compared model predictions with such *in vivo* measurements [4,7,11–13]. Curtis *et al.* [4] presented an MDA skull model of the lepidosaurian reptile *Sphenodon* (Rhynchocephalia) that produced comparable jaw movements and muscle activations with living animals. The bite forces predicted by this model, however, were consistently well below the *in vivo* forces [11]. To generate bite forces that match the *in vivo* values, Curtis *et al.* [11] had to increase the muscle forces they applied to their model by a factor of three. An MDA model by Moazen *et al.* [8] of the lizard *Uromastyx hardwickii* also predicted bite forces that were below measured bite forces in living animals with comparable skull dimensions [14].

There are a number of reasons why a computer model may underestimate bite force as the results of MDA depend on several input variables such as model geometry, mass properties, joint mobility or muscle force magnitudes and directions. Curtis *et al.* [11] suggested that inaccurate estimation of maximum muscle forces (e.g. owing to inaccurate values of muscle fibre length, muscle strength or the failure to consider the effects of pennation in highly pennate muscles) might explain the mismatch they found between predicted and measured bite forces. The accurate determination of maximum muscle forces was complicated in the study by Curtis *et al.* [11], because muscle measurements, model geometry and bite force measurements were obtained from different individuals. Therefore, differences in muscle size and bite forces had to be scaled using the limited baseline data available [14,15].

The extent to which model predictions are affected by inaccurate estimates of muscle forces can be assessed with sensitivity analyses. Recently, several elaborate sensitivity studies have been published that evaluated the relative importance of input variables in finite-element models of the feeding apparatus [16–20] and the results of these studies suggest that models are very sensitive to alterations of some variables. However, to date, there are fewer sensitivity studies for MDA skull models [10,12], so that the relative importance of different input variables for such models is largely unknown.

Here, we present the validation of an MDA skull model of the lizard *Tupinambis merianae*. This taxon is a large teiid lizard from South America with an omnivorous diet that includes plant material, ants, vertebrates and molluscs [21]. It was chosen because this study is the first part of a larger project on cranial kinesis in lizards where we compare taxa with different skull morphologies and kinetic potential. *Tupinambis* is particularly interesting for comparisons with monitor lizards (*Varanus*) from Africa, Asia and Australia. *Tupinambis* is unrelated to *Varanus*, but seems to be its ecological counterpart in the New World as these two taxa share many similarities in body shape, habit and feeding behaviour [22,23].

For our validation study, we use similar methods to those of Curtis *et al.* [4,11], but in contrast to these studies, all our data (anatomical data, bite force measurements, muscle force estimates and the image data used for the model geometry) were obtained from the same individual to eliminate inter-individual differences as a potential source of error. In addition, we conducted a sensitivity analysis, in which we assessed the effects of inaccuracies in the estimation of maximum muscle forces and muscle geometry on the model predictions.

## 2. Material and methods

### 2.1. Anatomical data

**Figure 1.**
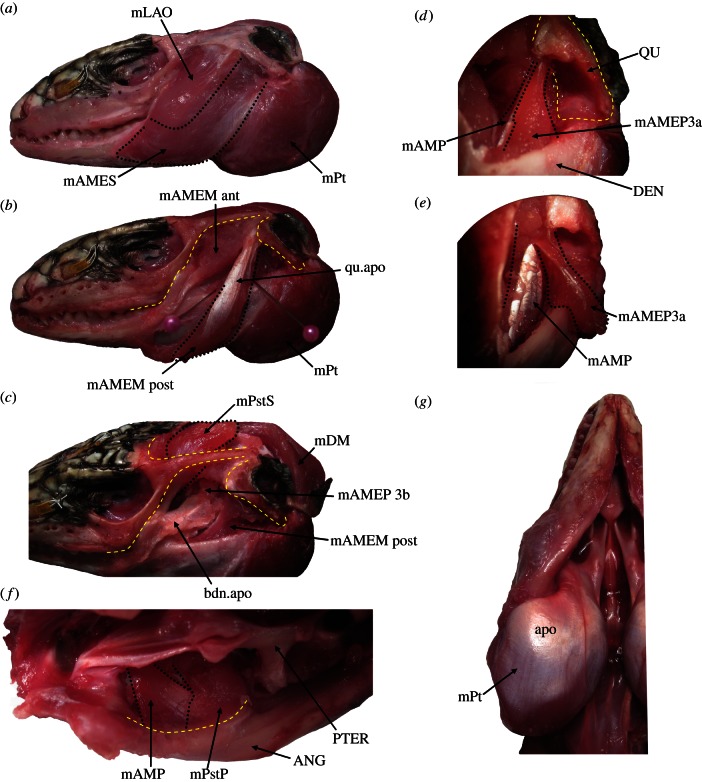
Dissections of *Tupinambis* T1 specimen: (*a*) lateral view with skin removed; (*b*) lateral view with the *m. adductor mandibulae superficialis* removed; (*c*) lateral view with the *m. adductor mandibulae superficialis* and most of the *m. adductor mandibulae medialis* removed; (*d*) anterolateral view of the lower temporal fenestra with the *m. adductor mandibulae medialis* removed; (*e*) as *d* but with the *m. adductor mandibulae profundus* 3*a* folded outward; (*f*) insertions into the adductor fossa of the lower jaw in ventromedial view after removal of the *m. pterygoideus*; (*g*) the *m. pterygoideus* in ventral view. ANG, angular; apo, aponeuroses; bdn.apo, bodenaponeurosis; DEN, dentary; mAMEM ant, *m. adductor mandibulae medialis* anterior part; mAMEM post, *m. adductor mandibulae medialis* posterior part; mAMES, *m. adductor mandibulae superficialis*; mAMEP3a, *m. adductor mandibulae profundis 3a*; mAMP, *m. adductor mandibulae posterior*; mDM, *m. depressor mandibulae*; mLAO, *m. levator anguli oris*; mPstS, *m. pseudotemporalis superficialis*; mPstP, *m. pseudotemporalis profundis*; mPt, *m. pterygoideus*; PTER, pterygoid; QU, quadrate; qu.apo, quadrate aponeurosis.

**Figure 2.**
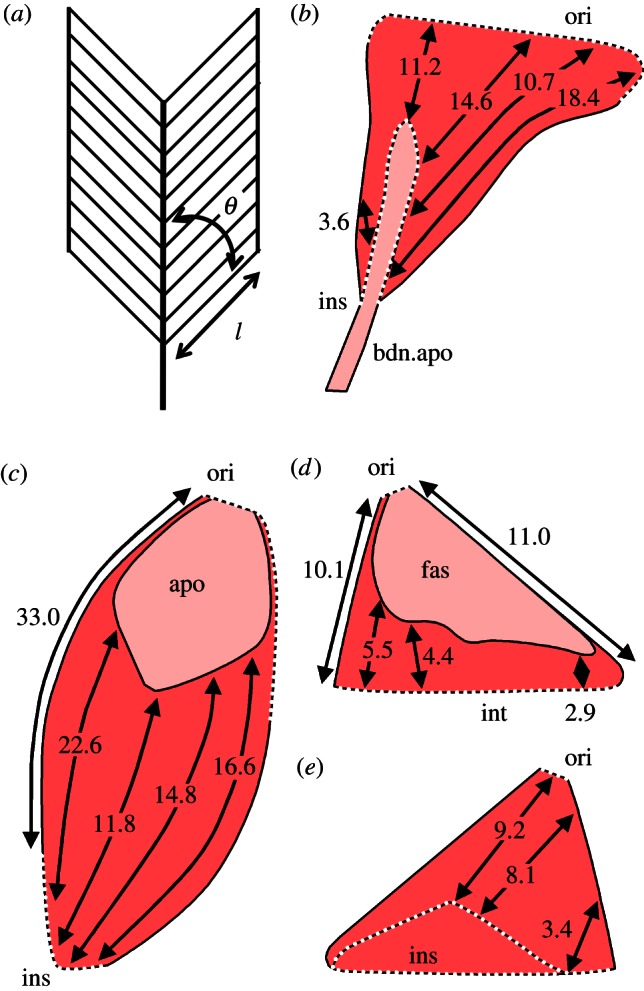
Schematic diagrams to illustrate variation in fibre length: (*a*) the measurements taken for the estimation of maximum muscle forces. Thick lines represent tendon, thin lines the borders between fascicles; *θ*, pennation angle; *l,* fibre or fascicle length. (*b*) *M. pseudotemporalis superficialis* in lateral view; (*c*) *m. pterygoideus* in ventral view; (*d*) *m. adductor mandibulae posterior* in lateral view; (*e*) *m. adductor mandibulae posterior* in medial view (*b–e*, all right side). apo, aponeurosis; bdn.apo, bodenaponeurosis; ins, insertion; fas, fascia; ori, origin. (Online version in colour.)

Two adult specimens of *T. merianae* were dissected under a binocular microscope to record details of muscle organisation, origin and insertion (figures 1 and 2). Fibre lengths, pennation angles and muscle weights were also collected from the specimen to be modelled (T1) following the procedure described in Anapol & Barry [24].

### 2.2. Bite force measurements

*In vivo* bite forces were recorded for T1 (an adult female, skull length = 88 mm, snout–vent length = 360 mm) housed at the University of Antwerp, Belgium. The measurements were taken with a piezoelectric isometric Kistler force transducer (9311B; range ± 5000 N, Kistler, Switzerland) at two different positions: (i) at the front of the jaw using the premaxillary teeth and (ii) further back on the tooth row, approximately halfway between the most anterior and the most posterior tooth. The measurements at each bite position were repeated 10 times, and the highest measured force from those trials was retained as a measure for maximum bite performance at each position [25,26].

### 2.3. Model construction

After the *in vivo* experiments, which included not only bite force measurements, but also a kinematic analysis, strain gauge measurements and electromyography (unpublished data), the individual T1 was euthanized. Subsequently, the head was scanned with an X-Tek HMX 160 micro-computed-tomography (µCT) scanner (X-Tek Systems Ltd, Tring, UK). The primary reconstruction resulted in a scan with a voxel size of 0.121 mm in all three axes. Based on these µCT data, three-dimensional models of the cranium and mandible were created in Avizo v. 6.3.1 (Visualization Sciences Group, USA) and imported to the multi-body dynamics software Adams 2011 (MSC Software Corp., USA).

The resulting skull model consisted of five rigid bodies: the cranium, the two quadrates and the two hemi-mandibles. Apart from the cranium, which was fixed, these parts could move independently and were connected to each other by different types of joints. The hemi-mandibles were connected at the mandibular symphysis by a spherical joint with 6 degrees of freedom (d.f.). The quadrate–mandibular joint was defined as a hinge joint (1 d.f.) and the quadrato-squamosal joint was defined as a spherical joint with 4 d.f. (constrained in the medial and posterior direction). These joint types and constraints were chosen based on the joint mobility measured during the dissection of the modelled individual. The mass properties of all moving parts were calculated in Adams based on the geometry of each rigid body and using a standard tissue density of 1.05 g cm^−3^ [12].

**Figure 3.**
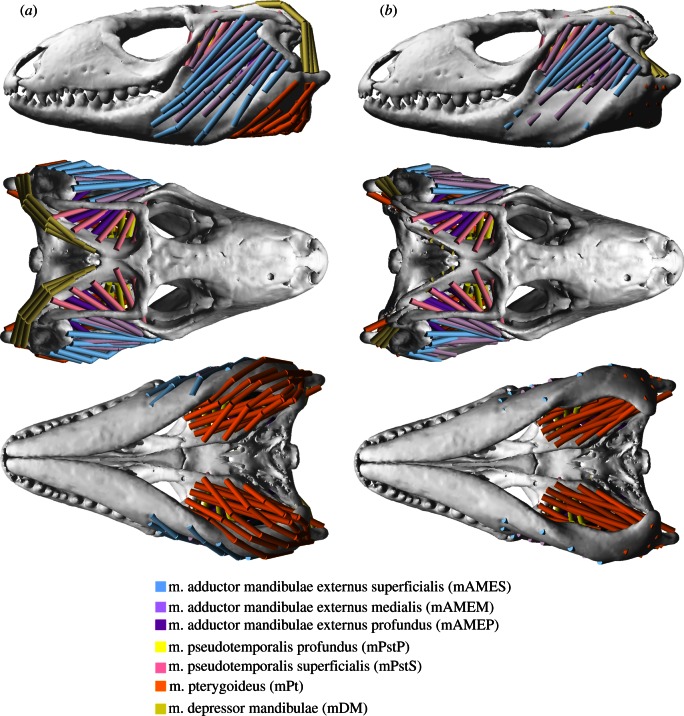
Multi-body computer models of the *Tupinambis* specimen (T1) used: (*a*) model with muscle wrapping; (*b*) model without muscle wrapping. The coloured cylinders represent muscle strands, which are modelled as springs. Note that the *m. adductor mandibulae posterior* (mAMP) is not visible in these views as this muscle is deep to the *m. pterygoideus* (mPt). Wrapping was applied to the following muscles: mAMES, mAMEM, mAMEP, mPt and mDM.

Muscle strands were included in the model as springs according to the observed origins and insertions of the main masticatory muscle groups. In one model, the muscle strands were modelled as straight lines connecting the origin and insertion sites. In a second model, some of the muscle strands were wrapped around the bone to model the orientation of the strands as accurately as possible (figure 3). By comparing the results from the model with and the model without wrapped muscles, the effect of muscle wrapping on model predictions was assessed. No attempt was made to include the complexities of the tendinous aponeuroses here. In addition, we did not include neck muscles in our model as *Tupinambis*, unlike other lizards such as *Tiliqua*, does not use head depression to increase bite forces. The final model included 116 muscle strands (58 on each side).

### 2.4. Multi-body dynamics modelling

The activation of these muscle strands during jaw opening and closing was modelled using dynamic geometric optimization, an algorithm that calculates muscle forces based on the orientation of the muscle strands to produce specific motions ([4] and electronic supplementary material, appendix S1). As a target motion, we used *in vivo* kinematic data, obtained from X-ray videos of the same individual during biting [27].

### 2.5. Estimation of maximum muscle forces

To estimate the peak force for each muscle, we used standard equations for physiological cross-sectional area (PCSA) and maximum muscle force ([24] and electronic supplementary material, appendix S1). These equations require a number of input variables that describe the biomechanical attributes of the modelled muscles: muscle mass, pennation angle in pennate muscles, fibre or fascicle length, the specific density and the intrinsic strength of muscle (see electronic supplementary material, appendix S1).

While muscle mass, fibre length and pennation angle were measured in the modelled specimen, the intrinsic strength and specific density of skeletal muscle were taken from the literature. For specific density, we used a value of 1.0564 g cm^−3^, which is based on measurements of the cat soleus muscle *in situ* [28]. Most published values for intrinsic strength vary between 25 and 40 N cm^−2^ [29–31]. We, therefore, applied three different intrinsic strength values to our model: 25 N cm^−2^ [31], which is an average value for different mammalian muscles, 32 [29] and 40 N cm^−2^ [30], which are both based on human jaw muscles. Using these values, we studied the sensitivity of the model predictions to the variation in intrinsic strength. In addition, we calculated maximum muscle force values from different fibre length estimates to take into account the uncertainty of these measurements. For each muscle, we calculated maximum muscle force for the minimum, maximum and average fibre length and assessed the model's sensitivity to these differences in muscle force values.

We also modelled the small passive tension that exists in each muscle strand. As muscles elongate, they offer a resistance (i.e. passive tension), which during jaw opening is a resistance in the jaw-closing muscles that the jaw-opening muscles must overcome (and *vice versa* during jaw closing). A maximum value of 0.15 N for this passive force has been used previously for a lepidosaur [4,11], but this maximum value was too large and impaired jaw opening in *Tupinambis*. A lower value of 0.10 N was chosen because it allowed complete jaw opening and resulted in maximum force values for *m. depressor mandibulae* that were close to the maximum muscle force value based on the PCSA values. This passive tension does not, however, affect bite forces and muscle activations during MDA biting simulations.

**Figure 4.**
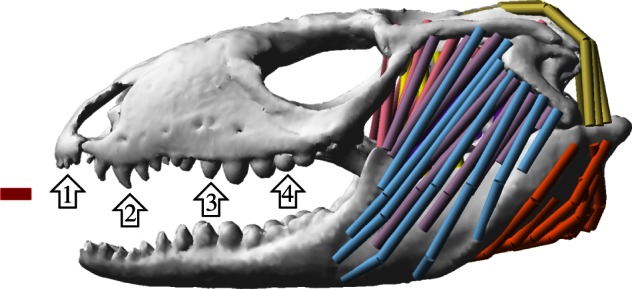
The four bite positions used for the comparison between measured and predicted bite forces. The red rectangle represents the bite force transducer. Each bite was modelled in an individual analysis.

We simulated unilateral biting at four different positions (figure 4). The bite force transducer was modelled as a rigid rectangular object. Apart from bite position 1, a symmetric bite with the premaxillary teeth, all bites were simulated on the left and the right side, and the average bite force value for each position was used for comparisons with the *in vivo* bite forces.

## 3. Results

### 3.1. Anatomical data

Our observations were generally consistent with previous descriptions [22,32,33] but direct examination provided data with a level of detail unobtainable by other means. The muscles include four external adductors, three internal adductors, the posterior adductor and the depressor mandibulae.

The *m. levator anguli oris* is a thin, sheet-like muscle that originates from the upper temporal bar and associated quadrate fascia (figure 1*a*), and inserts into the rictal fold (the fold of skin at the corner of the mouth). The underlying *m. adductor mandibulae externus superficalis* (mAMES) is, by contrast, the heaviest of the external adductor muscles (table 1). The mAMES originates from the upper temporal bar, the anterior edge of the quadrate and the lateral surface of the quadrate aponeurosis (a long tendon T-shaped in cross section, extending from the quadrate). It has a broad insertion on the lateral surface of the dentary (figure 1*a*). The deeper and closely associated *m. adductor mandibulae externus medialis* (mAMEM) includes: anterior (‘pinnate portion’ of Rieppel [22]) and posterior parts (figure 1*b*). The anterior part originates from the upper temporal bar and inserts on the bodenaponeurosis, a tendinous structure attached to the coronoid process of the lower jaw (figure 1*c*). The posterior part originates from the quadrate and quadrate aponeurosis, and inserts on the crest [22] and lateral surface of the dentary (*contra* [22]). Both the mAMES and mAMEM wrap around the lower jaws before insertion. The *m. adductor mandibulae externus profundis* (mAMEP) has three parts: the 3a part (*sensu* [22]) originates from near the head of the quadrate and inserts into the bodenaponeurosis [22] but also into the lateral part of the adductor fossa of the lower jaw (figure 1*e,f*). The 3b part originates from behind the supratemporal–parietal bar which it wraps underneath, and the 3c part originates from the lateral surface of the prootic. Both insert into the posterior portion of the bodenaponeurosis (figure 1*c*).

**Table 1.** Values used to calculate the physiological cross-sectional area (PCSA) and the maximum muscle force of each muscle group. The measurements were taken on the same individual as used in the computer model and the *in vivo* experiments.

muscle	muscle strands	weight (g)	angle of pennation (°)	fascicle length (mm)			PCSA (cm²)^a^	max. force (N)^b^		
				min.	max.	mean		25 N cm^−2^	32 N cm^−2^	40 N cm^−2^
mAMEMant	3	0.7	0–10	9.9	14.5	12.2	0.6	15.1	19.3	24.2
mAMEMpost	9	1.0	0	9.7	12.2	10.8	1.0	24.5	31.3	39.1
mAMEP3a	3	0.2	40	4.8	10.1	8.2	0.2	4.9	6.3	7.9
mAMEP3b	5	1.1	10–20	5.2	10.8	7.9	1.4	35.5	45.5	56.8
mAMEP3c	3	0.7	5–15	10.3	10.6	10.4	0.7	17.5	22.4	28.0
mAMES	7	3.3	25–45	8.6	23.4	15.9	1.8	45.0	57.6	72.0
mAMP	3	0.5	0–40	2.9	11.0	6.6	0.8	18.8	24.1	30.1
mPstP	4	1.7	0–5	7.0	25.1	18.4	1.0	24.4	31.2	39.0
mPstS	5	1.7	0–20	3.6	18.4	11.9	1.5	37.2	47.6	59.4
mPt	13	14.0	15–40	11.8	33.0	18.7	7.0	175.9	225.1	281.4
mDM	3	0.6	0–5	3.6	19.6	12.9	0.5	12.3	15.7	19.6

^a^Calculated using mean pennation angles and fascicle lengths.

^b^Calculated using three different values for intrinsic muscle strength (25, 32 and 40 N cm^−2^).

The *m. pseudotemporalis superficialis* (mPstS) originates from the embayed lateral surface of the parietal and inserts into a dorsal extension of the bodenaponeurosis (figure 1*c*). Deep to this the sheet-like *m. pseudotemporalis profundus* originates from the lateral edge of the parietal and the epipterygoid and inserts into the anteromedial edge of the adductor fossa (figure 1*f*). The *m. pterygoideus* (mPt) is easily the heaviest muscle and appears cushion-like (figure 1*g*). It originates from the pterygoid and inserts on the posteromedial and posterolateral surfaces of the lower jaw. Longer external fibres wrap around the shorter internal ones. The *m. adductor mandibulae posterior* (mAMP) originates from the medial part of the quadrate and inserts within the medial half of the adductor fossa (figure 1*e,f*), extending well forward into the Meckelian canal. Some fibres from the mPstS insert onto the tendinous sheet that forms the medial surface of the mAMP (figure 1*f*). The *m. depressor mandibulae* originates from a shelf on posteromedial surface of the parietal, wraps over the posterior tips of the parietal and inserts along the posterolateral edge of the retroarticular process of the lower jaw (figure 1*c*).

Differences in fibre length are apparent within the jaw muscles described earlier and these are often related to their pennate arrangement in relation to aponeurotic sheets (figure 2). For this reason, the values given in table 1 should be regarded as general estimates.

### 3.2. *In vivo* and modelling comparisons

**Figure 5.**
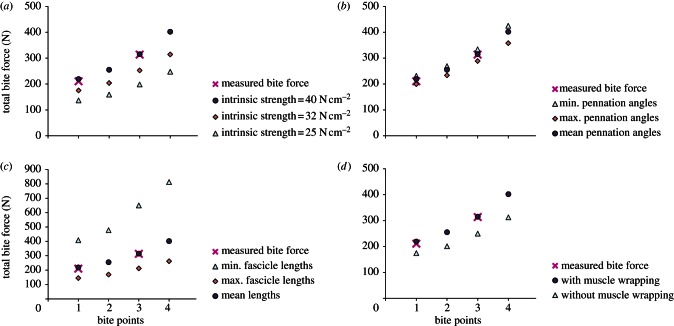
Measured and predicted bite forces for the bite point positions shown in figure 4. Four model variables were altered for sensitivity analyses: (*a*) the value used for intrinsic muscle strength; (*b*) the estimates of pennation angles by using the minimum, maximum and average estimates for each muscle; (*c*) the estimates of muscle fibre lengths by using the minimum, maximum and average estimates for each muscle; (*d*) muscle geometry by using the model with and without muscle wrapping. One variable was altered each time, whereas the others were kept constant: (*a–c*) all analyses with muscle wrapping; (*a,b,d*) average estimate for fibre length in all analyses; (*b–d*) intrinsic strength value = 40 N cm^−2^ in all analyses. (Online version in colour.)

The *in vivo* bite force measurements yielded maximum values of 211 N for a front bite and 314 N for a lateral bite. These two values were used for the validation of the MDA model. The bite forces predicted by the model show considerable variation, but there is a consistent, almost linear increase in bite forces as the bite position is moved towards the back of the tooth row (figure 5 and electronic supplementary material, appendix S2). This applies to all analyses and is consistent with the *in vivo* measurements. In addition, the measured bite forces are within the range of values predicted by the MDA.

When different values for intrinsic muscle strength are used to calculate the maximum force generated by each muscle (25, 32 and 40 N cm^−2^), predicted bite forces consistently vary accordingly (figure 5*a*, using mean fibre lengths and muscle wrapping). Thus, bite forces for an intrinsic strength of 25 N cm^−2^ are approximately 40 per cent lower than those for an intrinsic strength of 40 N cm^−2^, which results in absolute bite force differences for the two intrinsic strength values from approximately 80 (anterior bite) to 150 N (posterior bite). The highest intrinsic strength value, 40 N cm^−2^, yields the bite force predictions that come closest to the measured forces: at bite position 3, the measured and predicted bite forces are almost identical; at bite position 1, the predicted bite force is only approximately 8 N (4.0%) higher than the measured bite force.

There is some uncertainty regarding the exact position at which the maximum lateral bite force was measured during the experiment, either at bite point 3 or one tooth posterior to it, but the difference in bite force between these cases should be minimal because of the close proximity of the teeth.

The pennation angles used in the PCSA calculations have only a small effect on bite force predictions (figure 5*b*, using an intrinsic strength value of 40 N cm^−2^, mean fibre lengths and muscle wrapping). With minimum pennation angles predicted, bite forces are approximately 15 per cent (approx. 30 and 70 N for anterior and posterior bite positions, respectively) higher than with maximum pennation angles.

Muscle fibre length has a much larger effect on bite force predictions than pennation angles (figure 5*c*, using an intrinsic strength value of 40 N cm^−2^, mean pennation angles and muscle wrapping). The predicted bite forces are highest for minimum fibre length values and lowest for maximum fibre length values, because PCSA is inversely proportional to fibre length (see electronic supplementary material). The resultant variation in bite force magnitudes for different fibre length estimates is large: the predicted bite forces for maximum fibre lengths are approximately 65–70% lower than those for minimum fibre lengths, which leads to absolute bite force differences from approximately 260 to 550 N for anterior and posterior bite positions, respectively. Although different intrinsic strength values simply result in a linear scaling of the bite forces, different estimates for fibre length in each muscle affect the relative muscle forces among different muscles so that the changes in bite forces are nonlinear. Using an intrinsic strength value of 40 N cm^−2^, the bite force predictions come closest to the experimental values when mean fibre lengths are used.

Alterations of the orientations of the muscle strands also have a large effect on model predictions (figure 5*d*). The model without wrapped muscles predicts bite forces that are approximately 20 per cent lower than those predicted by the model with wrapped muscles, with the absolute bite force differences between the two models ranging between 45 and 90 N for anterior and posterior bite positions, respectively. Thus, bite forces are underestimated by the model without muscle wrapping.

## 4. Discussion

Our results highlight the importance of using subject-specific data for accurate modelling and demonstrate the value of conducting comprehensive sensitivity analyses to assess the effects of uncertainties and errors in the choice of input variables.

The degree to which the model predictions match experimental data is highly dependent on how the muscles are represented. Our sensitivity analyses show that changing pennation angles has a much smaller effect on predicted bite force magnitudes than does changing muscle fibre length or intrinsic strength (figure 5*a–c*). This is to be expected because the latter variables directly impact on maximum muscle force. Altering the orientation of the modelled muscle strands, and thus the muscle force vectors also affects predicted bite force magnitudes (figure 5*d*). In our model, muscle wrapping has a greater effect than extreme values of pennation angle but this is less than the effect of using extreme values of muscle fibre length.

Bite force predictions are more accurate when muscles are wrapped around bones as found in the real animal. The model without muscle wrapping underestimates *in vivo* bite forces by approximately 20 per cent. Therefore, although muscle wrapping is a time-consuming manual process, this effort is justified by its obvious benefits. In our model, a large number of muscle strands had to be wrapped around bones to simulate realistic muscle orientations. In some muscles, such as in the jaw depressors and the pterygoid muscles (figure 1), which are the most forceful muscle group of the jaw adductors in *Tupinambis* (table 1, mPt contributes 44% to the maximum force of all jaw adductors), the three-dimensional orientation of the muscle strands was altered dramatically by the wrapping, making the muscles’ lines of action more advantageous for jaw opening and closing.

That muscle wrapping has such a notable effect on bite force predictions is highly relevant for model building because many animals have jaw muscles wrapped around skull bones and other muscles [34–40]. Although often modelled as such, the path from origin to insertion is rarely linear. Because the degree of muscle wrapping differs between taxa and ontogenetic stages of the same taxon [37,39], the effect of muscle wrapping probably varies accordingly. Nevertheless, there is no reason to consider *Tupinambis* as an animal with particularly complex muscle wrapping. Rodents, primates, turtles and other squamates all demonstrate comparable or more complex muscle wrapping.

Besides the importance of accurate muscle geometry, our results stress the importance of obtaining accurate estimations for intrinsic muscle strength, fibre length and so maximum muscle force. However, the value for intrinsic strength is not known for the masticatory muscles of most species, including *T. merianae*. The intrinsic strength value that produces the bite force predictions closest to the *in vivo* data, 40 N cm^−2^, is at the upper end of the range that is typically found in the literature [29–31]. However, this value corresponds very well with measurements of the *m. adductor mandibulae externus superficialis posterior* in the lizard *Trapelus pallida* [41]. Based on these measurements, an intrinsic strength value of 43 N cm^−2^ (±10.9, *n* = 6) can be calculated. Our modelling results suggest that the intrinsic strength of jaw muscles in *T. merianae* is similar to that in *T. pallida*.

Unlike intrinsic strength, we were able to measure muscle fibre lengths in our modelled specimen. There are detailed descriptions on how to measure fibre lengths in the literature [24,42], but it is not straightforward. Complex pennate muscles such as those of the lepidosaurian adductor chamber are composed of numerous fibres of variable length inserting into a branching aponeurotic framework, so it is necessary to take several measurements in different parts of each muscle. This provides a general understanding of the pattern and limits of variation within each muscle. Measuring the length of every fibre is simply not feasible with current technology. High-resolution three-dimensional imaging of muscle, for example, with micro-magnetic resonance imaging [43] or iodine potassium iodide staining [44], could facilitate more accurate measurements in the future. Nevertheless, it is reassuring that in our study the averaged fibre length produced bite force predictions that were very close to the *in vivo* data. It seems that measurement errors were averaged out due to the fact that several measurements were taken in each muscle.

Compared with the variation in fibre length values, different estimates of fibre orientation have a much smaller effect on bite force predictions. This is not surprising because the use of cosines of angles in the formula used to calculate PCSAs (see the electronic supplementary material) means that potential inaccuracies in the measurement of fibre orientation or pennation angle will have a lesser effect on the results than potential inaccuracies in the measurement of fibre length when angles are near to 0, where the cosine function is relatively flat [11]. Most fibres have small pennation angles and this results in cosines that are close to one (e.g. a pennation angle of 20° returns a cosine of 0.94). Therefore, although measuring variation in fibre orientation is as challenging as measuring fibre length, accounting for it with complete accuracy is less crucial when angles are small.

As the validation of our model depends not only on the model predictions but also on the accuracy of the measured bite forces, we compared the measured bite forces of T1 (211 N for a front bite, 314 N for a lateral bite) with values from semi-wild animals of the same species [25]. The T1 values are close to but slightly lower than the values measured in semi-wild animals with comparable jaw length. This could be because the individual tested in this study lived on a different diet and thus might have less developed masticatory muscle mass compared with semi-wild animals. Indeed, unpublished data that have been collected by one of us, A.H., suggest that T1 has lower muscle masses and cross-sectional areas than the semi-wild animals. In addition, there are published bite force data available for *Tupinambis teguixin* [45], but those bite forces are not directly comparable to our values as the measurements in *T. teguixin* were taken while the lizards were fed mice, whereas our values are maximum bite forces during defensive bites.

We have shown that multi-body dynamic modelling can simulate skull mechanics in a realistic way if muscle forces are based on accurate measurements, and the geometry of muscles is modelled with fidelity. Our model predicts bite forces that closely match *in vivo* measurements. This contrasts with many previous biomechanical models using PCSA [8,46–50]. Previous MDA models of lizard and *Sphenodon* skulls [8,11] may have underestimated bite forces because of inter-individual differences, because the models and *in vivo* bite force data were not based on the same individuals. This potential source of error was avoided here by using the same individual for both the *in vivo* and modelling work. Thus, subject-specific data should be used wherever possible to ensure accurate prediction of bite force using MDA. However, in studies where absolute values are not of interest, but only gross or relative differences between species, subject-specific models may not be necessary if sufficient sensitivity analyses are conducted. This is especially important for studies of extinct taxa where subject-specific muscle force estimates are unavailable.
